# Ultrasound Evaluation of Plantar Fascia in Individuals with Charcot–Marie–Tooth Disease: An Exploratory Study

**DOI:** 10.3390/life15111741

**Published:** 2025-11-12

**Authors:** Noemi Vallario, Antonella Vitale, Alessandra Zeni, Eleonora Di Ciesco, Eloisa Martire, Rossella Calciano, Letizia Tarallo, Gianpaolo Ronconi, Costanza Pazzaglia, Ilaria Paolasso, Augusto Fusco

**Affiliations:** 1 Department of Geriatrics and Orthopaedics, Catholic University of the Sacred Heart, 00168 Rome, Italy; noemi.vallario@gmail.com (N.V.); alessandrazeni6@gmail.com (A.Z.); elinor96edc@gmail.com (E.D.C.); eloisa-m@hotmail.it (E.M.); rossella.calciano@gmail.com (R.C.); letizia.tarallo@gmail.com (L.T.); 2 UOSD Physical Medicine and Rehabilitation, Department of Aging, Orthopedic, and Rheumatological Sciences, University Hospital Foundation “Agostino Gemelli” IRCCS, 00168 Rome, Italy; gianpaolo.ronconi@policlinicogemelli.it; 3 UOSD High-Intensity Neurorehabilitation, Department of Neuroscience, Sensory Organs and Thorax, University Hospital Foundation “Agostino Gemelli” IRCCS, 00168 Rome, Italy; costanza.pazzaglia@policlinicogemelli.it (C.P.); ilaria.paolasso@policlinicogemelli.it (I.P.); 4 UOS High-Speciality Neurorehabilitation, Department of Neuroscience, Sensory Organs and Thorax, University Hospital Foundation “Agostino Gemelli” IRCCS, 00168 Rome, Italy

**Keywords:** Charcot–Marie–Tooth disease, plantar fascia, ultrasound, rehabilitation, functional outcomes

## Abstract

Background: Charcot–Marie–Tooth disease (CMTd) is the most prevalent inherited peripheral neuropathy, often associated with foot deformities and gait and balance impairments. While the structural characteristics of the foot have been extensively investigated, limited data are available regarding the features of the plantar fascia in individuals with CMTd. Aim: To investigate the ultrasound (US) structural characteristics of the plantar fascia in subjects with CMTd and to explore their relations with disease severity and functional outcomes, encompassing lower extremity function, gait, and balance. Methods: A total of 26 individuals with confirmed CMTd underwent clinical and functional assessments. Bilateral ultrasound examination of the plantar fascia was performed to assess thickness, echogenicity, fibrillar pattern, and inflammatory signs (as assessed by US Power Doppler). Correlations between ultrasound findings, clinical data, and functional measures were also evaluated. Results: No pathological increase in plantar fascia thickness was observed, although a significant side-to-side difference was noted (*p* = 0.031) on ultrasound (US) imaging. No inflammatory signs were also detected. Significant associations were found between fascial alterations and age (*p* = 0.024), disease severity (CMTES, *p* = 0.014), and functional performance (10 MWT *p* = 0.017; SPPB *p* = 0.039). Conclusions: In individuals with CMT, plantar fascia abnormalities likely reflect chronic structural degeneration rather than acute inflammation. These changes are more evident with an increase in age, disease progression, and functional decline, suggesting the role of US imaging as a valuable tool for clinical and therapeutic strategies.

## 1. Introduction

Charcot–Marie–Tooth disease (CMTd) and its related disorders represent a heterogeneous group of genetically determined peripheral neuropathies. CMTd is the most prevalent hereditary neuropathy, affecting approximately 1 in 2500 people [1].

The broad range of clinical manifestations and the heterogeneity in disease progression can be ascribed to the variability of the underlying nerve degeneration [2]. Initial symptoms usually manifest in the lower limbs and progress from the distal to the proximal segments over time. Common clinical features include pes cavus (high-arched feet), hammertoes, and foot muscle weakness [2,3]. Further manifestations may encompass muscle cramps, pain, and plantar calluses, resulting from abnormal walking patterns [4].

Foot deformities are observed in up to 74% of individuals with CMTd [5]. The hallmark cavus foot results from muscle imbalances, involving overactivity of the peroneus longus and tibialis posterior muscles, associated with weakness of the tibialis anterior and peroneus brevis muscles [6,7]. It has also been shown to exhibit increased forefoot supination and hindfoot malalignment compared to the idiopathic cavovarus foot [8].

In general, foot deformities are frequently accompanied by a thickened and contracted plantar fascia [7]. Inflammatory changes at the site of the insertion of the plantar fascia, in combination with a loss of tissue elasticity and a reduction in heel shock absorption, may lead to plantar fasciitis [7]. Individuals with pes cavus are more predisposed to plantar fasciitis than those with normal or flat feet [9]. This may be attributed to the fact that vertical and ground reaction forces can exert mechanical stress on the plantar fascia, potentially resulting in chronic overload due to excessive tension being applied [10]. The severity of these conditions can be exacerbated by inherited tissue deformities related to the etiopathogenesis of CMTd. In this condition, peripheral nerve dysfunction and the resulting muscle imbalances can alter loading patterns on the plantar fascia, potentially contributing to increased tension, stiffness, and foot discomfort.

Nevertheless, the role of the plantar fascia in CMTd remains largely unexplored. To date, no studies have specifically investigated the relationship between cavus foot deformities and plantar fascia features in individuals with CMTd. No specific examination of the plantar fascia characteristics has been conducted in these individuals. The investigation of the plantar fascia is fundamental for its role in supporting the plantar arch, stabilizing the foot, facilitating propulsion during gait, and absorbing mechanical shocks [11]. Ultrasound (US) imaging is particularly suitable for the study of the plantar fascia, for its accessibility, reliability, and cost-effectiveness [12,13].

The aim of this observational study was to describe the structural characteristics of the plantar fascia in subjects with CMTd at US imaging. In particular, a detailed analysis of US parameters (thickness, echogenicity, fibrillar pattern, and inflammation signs) has been conducted. As a secondary outcome, a subsequent analysis of the relation with disease severity and functional outcomes, encompassing lower extremity function, gait, and balance, was also performed.

## 2. Materials and Methods

### 2.1. Population and Study Protocol

This observational study was conducted in the outpatient department of an academic hospital. All participants were recruited from a specialized clinic dedicated to individuals with Charcot–Marie–Tooth disease (CMTd), where patients execute regular multidisciplinary visits. A total of 26 individuals with CMTd who volunteered to participate in the study, were enrolled over the course of the year 2024, from 2 January to 30 December. All participants had a genetically confirmed diagnosis of CMTd established by a neurologist, based on clinical, electrophysiological, and genetic testing performed prior to recruitment. All the participants presented with pes cavus consistent with their clinical features of the disease.

The eligibility criteria comprised specific inclusion and exclusion parameters, as detailed below.

Inclusion criteria:

• Adult individuals aged 18 years or older with a certified diagnosis of CMTd;
• Attendance at the CMTd ambulatory as part of routine clinical care;
• Ability to perform gait and standing balance assessments.

Exclusion criteria:

• Presence of other diagnosed peripheral neurological disorders;
• Lack of prior consent for the use of biological data for scientific purposes.

A detailed clinical history was collected for each participant, including demographic information, the presence of motor and sensory symptoms, gait disturbances, current physiotherapy involvement, participation in sports and physical activity, and use of assistive devices or orthoses.

The study was conducted in accordance with the principles of the World Medical Association Declaration of Helsinki. Written informed consent was obtained from all participants prior to the use of biological data.

### 2.2. Clinical Scales

In accordance with the study by Mori and colleagues on the assessment of functional impairment in CMT1A [14], all subjects were evaluated by using a standardized battery of the subsequent clinical scales, and prior to the US examination, performed one after the other, in the same order as follows:

- Charcot–Marie–Tooth Examination Score (CMTES) [15]: A clinician-administered scale based on a physical examination that measures motor and sensory impairments. This is a sub-score of the CMT Neuropathy Score [15]. The scale ranges from 0, as healthy status, to 28, indicating a severe condition. The assessment is based on both symptoms and clinical signs. The scale is employed for the purpose of disease severity classification, with the severity of the disease being categorized as follows: mild (score 0–7), moderate (8–14), or severe (>15).
- Tinetti Performance-Oriented Mobility Assessment [16]: A performance-based test used to evaluate balance and gait. Lower scores have been shown to be indicative of poorer balance and higher fall risk. It has previously been demonstrated that this scale is a valid instrument for subjects with CMTd [17], with a strong negative correlation between the Tinetti scale and the severity of the disease.
- Walk-12 Questionnaire: A self-reported measure, used to assess the perceived impact of neuropathy on walking ability [18]. Scores within this scale range from 0, indicating no limitation, to 60, indicating a severe limitation. The measure was originally developed as a disease-specific tool. Holland and colleagues demonstrated the reliability and sensitivity of the test in detecting walking impairments across various neurological conditions, including CMTd [19].
- Short Physical Performance Battery (SPPB): A composite test that evaluates lower extremity function through a series of assessments, including balance, gait speed, and the chair stand test [20]. Scores within this system range from 0 to 12, with higher scores indicating superior physical performance. A previous study has employed SPPB on subjects with CMTd, validating its reliability and relevance as an outcome measure in this population [21].
- 10 Meter Walk Test (10 MWT): A functional walking test in which participants are requested to walk 10 m at a self-selected pace [22]. The speed of walking is measured in meters per second (m/s).

### 2.3. Instrumental Evaluation

For each participant, a US examination of the plantar fascia of both feet was conducted by the same physician in our ward. The assessments were conducted with the same machine (HITACHI Preirus Hi Vision; Hitachi Ltd., Tokyo, Japan), by means of a 10–18 MHz linear-array transducer.

**Figure 1:**
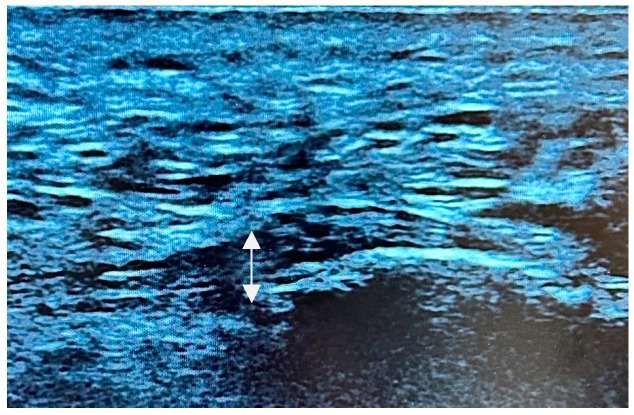
US assessment of the plantar fascia at the point where it is thickest near the calcaneus and its thickness measurement, shown by the white arrow.

The examinations were performed in accordance with a standardized protocol, with participants assessed in the prone position [23,24]. Due to the presence of the typical foot deformities, the foot was maintained in a neutral or slightly dorsiflexed position. This position was adopted to ensure appropriate tension to the fascia, thus optimizing its visualization for US assessment. The thickness of the plantar fascia was measured in B-mode, in millimeters (mm), along the longitudinal axis with a slight medial obliquity, at the point where the fascia was thickest near the calcaneus (see Figure 1). More specifically, the transducer was placed longitudinally along the plantar surface of the foot, starting at the medial calcaneal tuberosity, and extending distally towards the metatarsal heads [25].

The standard US appearance of the plantar fascia typically shows a thickness of 2–4 mm in healthy individuals [25,26,27]. Inflammation at the insertion site was evaluated using Power Doppler mode [28].

**Figure 2:**
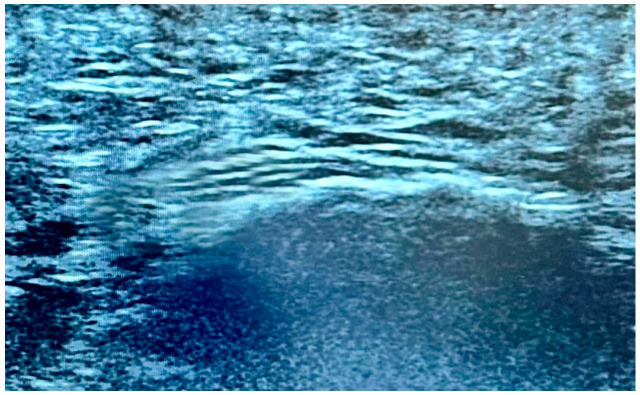
US image showing Type 1, characterized by a preserved fibrillar structure and no evidence of hypoechogenicity.

**Figure 3:**
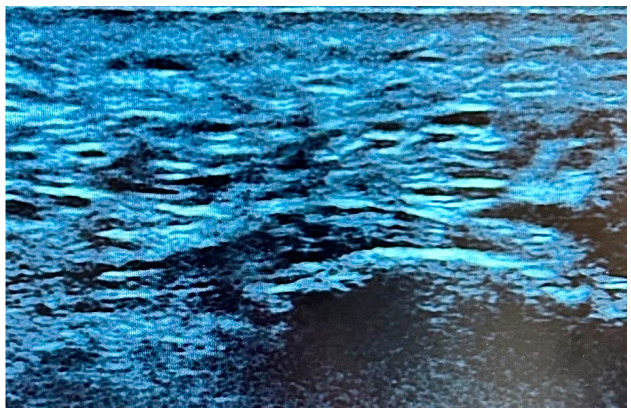
US image showing Type 2, with hypoechoic appearance of the plantar fascia and moderately disrupted fibrillar structure.

**Figure 4:**
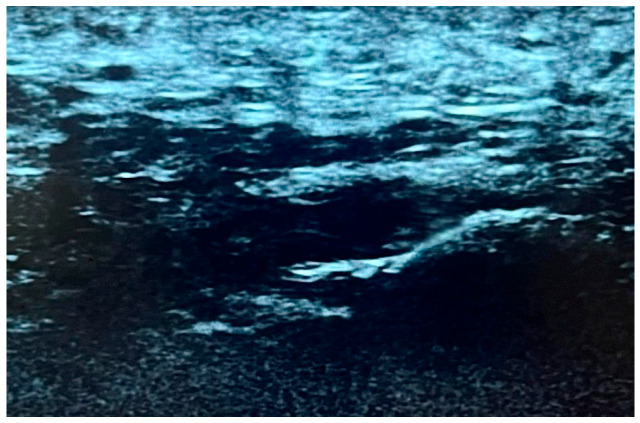
US image showing Type 3, displaying pronounced hypoechogenicity and a severely disrupted fibrillar structure.

In the absence of a scientifically validated classification system that incorporates both echogenicity and fibrillar pattern, a qualitative evaluation of the fibrillar pattern and echogenicity of the plantar fascia was conducted. Similar changes have been observed in other conditions affecting the plantar fascia [29]. A three-level grading system was proposed, based on the presence of hypoechogenicity and the extent of disruption of the fibrillar pattern. In this sense, our approach is consistent with the clinical classification of individuals with CMTd, which is commonly used to describe the severity of symptoms. Consequently, the US alterations observed in our cohort of enrolled subjects were categorized as follows:

- Type I: no presence of hypoechogenicity and preserved fibrillar structure (Figure 2).
- Type II: hypoechoic fascia with moderately disrupted fibrillar structure (Figure 3).
- Type III: hypoechoic fascia with severely disrupted fibrillar structure (Figure 4).

### 2.4. Statistical Analysis

The characteristics of participants were described using absolute frequencies and percentages for categorical variables and means with standard deviations for continuous variables. Similarly, descriptive analyses of clinical scale scores and ultrasound parameters were conducted.

To investigate the associations between age, sex, ultrasound findings of the plantar fascia (including fascia thickness and ultrasound pattern), and clinical scale scores, the following statistical tests were applied: the Shapiro–Wilk test was used to assess normality; the Wilcoxon signed-rank test and Kruskal–Wallis test were used for non-parametric comparisons; the *t*-test was used for parametric comparisons; the Spearman’s and Pearson’s correlation coefficients were used to evaluate continuous variables. Test selection was based on the distribution of the data and the parametric or non-parametric nature of each variable (see in Supplementary Materials).

Statistical analyses were performed using IBM SPSS Statistics (Version n. 25), setting the significance level at *p* < 0.05 for the main tests, and *p* < 0.025 for post hoc analyses (confidence interval at 95%).

## 3. Results

The study group consisted of 26 individuals diagnosed with CMTd, who underwent a US examination of both feet and a clinical evaluation (see Table 1 for demographic and clinical features of the sample).

Table 2 and Table 3 present the findings of the descriptive analysis of the plantar fascia US data and the clinical scales, respectively.

The statistical analysis revealed no significant differences in plantar fascia thickness for demographic variables such as age and sex. A statistically significant difference in plantar fascia thickness was observed between the right and left sides (*p* = 0.031; Paired *t*-Test). In addition, no significant differences were found in plantar fascia thickness between CMT subtypes and for the different clinical scales.

**Figure 5:**
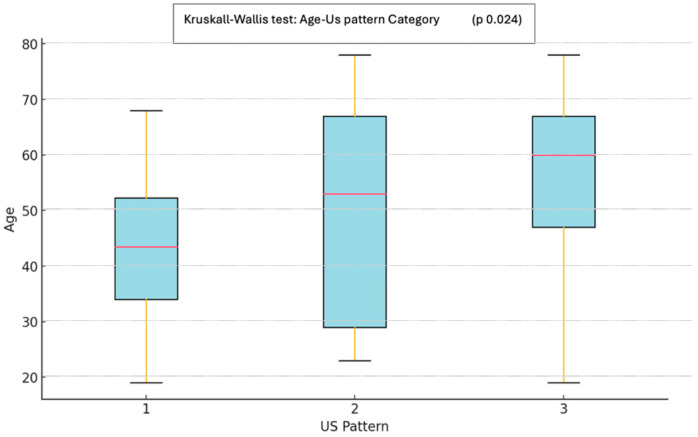
Whisker-plot of the three types of US pattern and age, resulting in a significant difference among the category (*p* = 0.024). Significant differences were found between type 1 and 3 (*p* = 0.024; Wilcoxon post hoc analysis).

The US pattern of the plantar fascia, categorized into three types based on structural alterations, showed no significant differences between genders. In contrast, a significant difference in age was observed across the three US patterns (*p* = 0.024; Kruskal–Wallis test), with more severe plantar fascia US alterations noted in older patients. Subsequent post hoc analysis revealed a significant difference between type 1 (absence of pathological US features) and type 3 (hypoechoic fascia with severely disrupted fibrillar structure), as illustrated in Figure 5.

**Figure 6:**
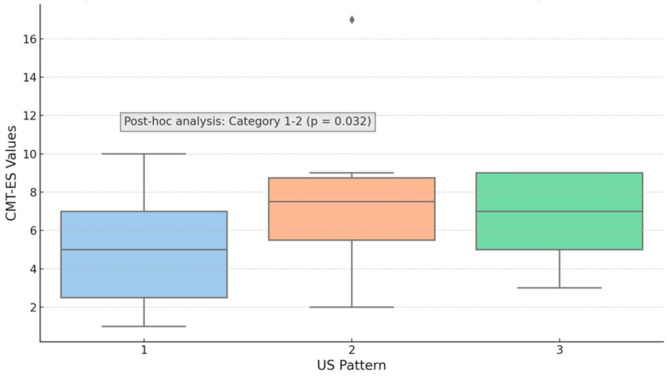
Whisker-plot of the three types of US pattern analyzed in terms of CMTES (*p* = 0.014; Kruskal–Wallis test). A *p*-value close to significant difference was found between type 1 and 2 (*p* = 0.032; Wilcoxon post hoc analysis).

Similar statistical differences in CMTES values were found among the plantar fascia pattern groups (*p* = 0.014; Kruskal–Wallis test). Post hoc analysis demonstrated a threshold *p*-value close to significance between types 1 and 2 (hypoechoic fascia with moderately disrupted fibrillar structure) (Figure 6). Despite the relatively small sample size, a mild positive correlation was found between plantar fascia degeneration and clinical severity (CMTES absolute values, Rho = 0.33, *p* = 0.017; Spearman’s rank correlation). These findings suggest that individuals presenting more severe clinical symptoms exhibit greater structural degeneration of the plantar fascia.

Finally, statistically significant results were observed between ultrasound pattern categories and both the 10 MWT (*p* = 0.017) and the SPPB (*p* = 0.039), showing significantly different scores on the 10 MWT between pattern categories 1–2 (*p* = 0.019) and 1–3 (*p* = 0.022), and on the SPPB between pattern categories 1–3 (*p* = 0.013). Analysis of mean scores across the groups of different US patterns showed that patients with more severe alterations showed lower walking speeds on the 10 MWT and lower SPPB scores, reflecting reduced physical performance. No statistically significant differences were found in the Walk12 Questionnaire (*p* = 0.126) and the Tinetti Performance-Oriented Mobility Assessment (*p* = 0.201).

## 4. Discussion

The present study investigated the ultrasound features of the plantar fascia in individuals with CMTd. Additionally, the obtained values were examined in relation to the most commonly used clinical and functional scales for this population. Although foot structural deformities represent a core clinical manifestation of the disease, it is noteworthy that the US characteristics of the plantar fascia have not been previously described. The results of this study may provide valuable insights into both the clinical evaluation and management of individuals with CMTd.

In our sample, all participants had cavus feet. However, no pathological alterations on the thickness of the plantar fascia were identified. The mean thickness of the right side was 3.2 mm, whereas the left side was 3.5 mm [25,26,27]. A significant difference was noted between right and left sides, possibly indicating limb dominance that may have influenced this result. Plantar fascia thickness was not associated with any demographic variables, including age and gender, as well as CMT subtypes. These results were consistent with the absence of US inflammatory markers, such as increased vascularization on Power Doppler [28]. It is possible that the use of shoe inserts may have mitigated signs of inflammation, as 42% of participants used some type of shoe insert. Noteworthy, none of the subjects enrolled in the study used AFOs (see Table 1).

Conversely, an altered structural pattern of the plantar fascia, with respect to echogenicity and fibrillar organization, was observed. Three types of US patterns, differing in echogenicity and fibrillar structure, were classified. Significant differences were identified for age (and not for sex) and for the severity of clinical impairment in CMTd. In particular, individuals with more severe clinical presentations, as indicated by higher CMTES, exhibited more altered fascial US features. Furthermore, significant associations were found between poorer ultrasound patterns and lower performance in functional assessments, including the 10 MWT and SPPB, suggesting that structural degeneration and motor impairment may be linked.

These findings support the hypothesis of a chronic degenerative process, probably related to biomechanical alterations observed in CMTd, including medial shifting of the talar head, which contributes to increased varus alignment, internal rotation, and adduction at the subtalar and talonavicular joints [30,31]. These changes have been shown to exacerbate the cavovarus deformity, resulting in a stiffer foot structure with reduced shock absorption that requires compensatory gait adaptations [31].

The US alterations in our study may be associated with changes in gait characteristics in subjects who had poorer functional outcomes, as determined by CMTES and 10 MWT and SPPB assessments. These results are clinically consistent with those of Park and colleagues [32], who demonstrated that poorer gait parameters are associated with more severe CMTd. In comparison to healthy subjects and mild patients, patients with moderate disease demonstrated greater hip flexion angles during the swing phase and smaller dorsiflexion angles at initial contact during the stance phase. In addition, children and adolescents with CMTd exhibit early kinematic and kinetic gait abnormalities, including significant foot drop during the swing phase, reduced calf muscle power, and proximal compensatory mechanisms in the lower limb [33]. Different studies have demonstrated as reduced ankle dorsiflexion is the most significant risk factor for the onset of plantar fasciitis [34,35]. In addition, structural modifications (function and/or anatomy) have been shown to negatively affect the performance of more complex functional tasks, such as running, as evidenced by conditions including, but not limited to, cerebral palsy [36].

In CMTd, distal muscle weakness, imbalance, and altered biomechanics can disrupt the function of this interconnected system, increasing strain along the plantar fascia–Achilles–lower leg fascial chain. These changes can impair upright posture and gait, and are associated with features such as cavus foot, ankle instability, and fatigue. The absence of significant differences in other functional evaluations (Tinetti Performance-Oriented Mobility Assessment and Walk-12 Questionnaire) may be related to the heterogeneity of our study sample (ambulatory; observational study) and the relatively small sample size, even though a slight correlation was observed between changes in US plantar fascia structure and Walk-12 results.

US imaging could be easily integrated with clinical data derived from functional scales in individuals with CMTd. Its use facilitates non-invasive longitudinal monitoring, which is particularly advantageous in the context of chronic diseases such as CMTd. Our data may be useful for clinical applications, as the plantar fascia can be a target for therapeutic interventions (surgery, orthoses, conservative treatments including rehabilitation). Due to the crucial role of the plantar fascia in gait and balance, early interventions aimed at preserving its integrity could mitigate disease progression, reduce fall risk, improve mobility, and promote independence and social participation. These interventions may also contribute to the clinical management of patients, indicating the need for conservative treatments and the necessity and eventually the timing of the surgical treatments. Moreover, it has been demonstrated that the plantar fascia may be considered a predictor of the development of complications such as peripheral neuropathy in diabetes [37].

Our results should be evaluated in light of the study’s limitations, including the relatively small sample size and the limited representation of CMT subtypes other than CMT1A. Furthermore, no healthy individuals were enrolled as a control group due to the exploratory and observational nature of our study, with no interventions. At the same time, no similar studies had been conducted before. We note that it cannot be methodologically stated whether these changes in the fascia are specifically associated with the disease or determined by age. Although age-related differences (sarcopenia, alterations of the soft tissue determined by age, etc.) may exist, all analyses were adjusted for disease severity and functional outcomes, more directly linked to the neuropathic process. Due to the limited sample size and the exploratory nature of this study, further stratification by age reduced the interpretability of the data. As well, it cannot be determined whether severe structural deformities of the feet and the ankles in the subjects with CMTd have determined an initial hypothesis of similar observations in their related tissues, and, in particular, in the plantar fascia. Moreover, the US evaluation was restricted to the insertion site of the plantar fascia, in accordance with standard clinical practice, without completing the assessment along its entire length. A more extensive ultrasound examination of the plantar fascia could have provided additional valuable insights into the structural changes associated with CMTd. Future studies are necessary to evaluate more sites of assessment in the plantar fascia at a US imaging. Finally, although we proposed and applied a grading system combining echogenicity and fibrillar pattern to improve assessment consistency, this approach remains unvalidated and potentially subject to observer bias. In next studies, the use of validated image analysis tools (e.g., ImageJ) to quantify echogenicity based on grayscale percentage or comparable parameters could strengthen the reliability of the US-reported data, also adding valuable information for clinical practice. In addition, other clinical elements such as pain symptoms, the presence or absence of heel spurs, and other US signs of plantar disease or more specific elements of the gait pattern were not investigated. In our findings, a significant difference was noted between the right and left sides in terms of thickness of the plantar fascia. No specific analysis of limb dominance was performed during the clinical evaluation phase, due to the exploratory nature of the study with a relatively small sample size. Our results need to be confirmed in future larger studies, also including the assessment of the dominance of the lower limbs with clinical/instrumental tests or scales.

Future studies should aim to recruit a larger and more heterogeneous patient cohort, encompassing a broader spectrum of CMT subtypes and follow them longitudinally over several years, possibly with an analogous control group. Increasing the sample size could allow the use of multivariate analysis by use of a correct power analysis in statistics before the study design, adding interpretive value to clinical variables that may be associated with plantar fascia parameters. This would provide a more comprehensive understanding of the natural history of the disease and its response to therapeutic interventions, with a particular focus on ultrasonographic changes in the plantar fascia following conservative treatments and/or the use of shoe inserts or other types of orthoses, and their potential impact on functional impairment [38].

## 5. Conclusions

Based on our findings, a greater clinical severity is associated with a poorer plantar fascia echostructure. Additionally, degraded fascia US characteristics are significantly associated with reduced functional performance. Qualitative findings of US features (echogenicity and fibrillar pattern) are more likely to be associated with clinical impairments than thickening. Due to the chronic nature of the disease and the need for regular, long-term monitoring, this exploratory study supports the usefulness of ultrasound imaging as a non-invasive diagnostic tool for monitoring patients with CMTd. The preliminary findings of this study highlight as further imaging studies involving a larger sample size are required in order to assist clinicians in better supporting the clinical evaluation and decision-making process.

## Figures and Tables

**Table 1:** Summary of demographic and clinical features of the enrolled individuals.

**Age (Years)**	Mean (SD)	48.6 (16.0)	
	Min–Max	19–78	
**Sex (n)**	Female	14	53.9%
	Male	12	46.1%
**CMT genetic type (n)**	CMT1A	17	65.5%
	CMT2	2	7.7%
	CMTX1	1	3.8%
	CMT4	1	3.8%
	HNPP	1	3.8%
	Ongoing diagnoses	4	15.4%
**Motor symptoms (n)**	Yes	23	88.5%
	No	3	11.5%
**Sensory symptoms (n)**	Yes	23	88.5%
	No	3	11.5%
**Walking difficulty (n)**	Safe environment	6	23.1%
	No-safe environment	9	34.6%
	No difficulties	11	42.3%
**Physiotherapy (n)**	Yes	4	15.4%
	No	22	84.6%
**Regular exercise (n)**	Sport	1	3.8%
	Gym fitness activities	8	30.8%
	Home stretching	8	30.8%
	No activities	9	34.6%
**Walking aids**	Yes	3	11.5%
	No	23	88.5%
**Use of orthoses**	Shoe-insert	6	23.1%
	Shoe-insert + orthopedic shoes	5	19.2%
	AFO	0	0%
	No	15	57.7%

SD: Standard Deviation; AFO: Ankle-Foot-Orthosis.

**Table 2:** Descriptive analysis of plantar fascia ultrasound data.

Parameter	Right	Left
**Plantar fascia thickness (mm)**		
Mean (SD)	3.2 (0.6)	3.5 (1.0)
Min–Max	2.1–4.2	2.1–6.7
**Power Doppler**		
Presence/Absence (%)	3.8%/96.1%	7.7%/92.3%
**Ultrasound Pattern** Type (%)	Type I: 57.7%	Type I: 46%
	Type II: 27%	Type II: 27%
	Type III: 15.3%	Type III: 27%

SD: Standard Deviation.

**Table 3:** Descriptive analysis of clinical scale results.

Scale	Mean (SD)	Min–Max
**CMTES**	6.1 (3.4)	1–17
**Tinetti**	21.5 (5.2)	10–28
**10 MWT**	0.8 (0.2)	0.4–1.1
**SPPB**	6.5 (3.0)	1–10
**WALK12**	34.5 (13.7)	12–59

SD: Standard Deviation.

## Data Availability

The original contributions presented in this study are included in the article/Supplementary Materials. Further inquiries can be directed to the corresponding authors.

## Supplementary Materials

The following supporting information can be downloaded at: https://www.mdpi.com/article/10.3390/life15111741/s1, Excel File: DB-raw data.

## Abbreviations

The following abbreviations are used in this manuscript:

CMT	Charcot–Marie–Tooth
CMTd	Charcot–Marie–Tooth disease
US	Ultrasound
CMTES	Charcot–Marie–Tooth Examination Score
SPPB	Short Physical Performance Battery
10 MWT	10 Meter Walk Test
